# Intracellular replication of *Pseudomonas aeruginosa* in epithelial cells requires suppression of the caspase-4 inflammasome

**DOI:** 10.1101/2023.02.13.528260

**Published:** 2023-02-13

**Authors:** Abby R Kroken, Keith A Klein, Patrick S Mitchell, Vincent Nieto, Eric J Jedel, David J Evans, Suzanne M J Fleiszig

**Affiliations:** 1 Herbert Wertheim School of Optometry & Vision Science, University of California, Berkeley, CA USA; 2 Department of Microbiology and Immunology, Loyola University Chicago, Maywood, IL USA; 3 Department of Microbiology, University of Washington, Seattle, WA, USA; 4 College of Pharmacy, Touro University California, Vallejo, CA USA; 5 Graduate Groups in Vision Sciences, Microbiology, and Infectious Diseases & Immunity, University of California, Berkeley, CA USA

## Abstract

Pathogenesis of *Pseudomonas aeruginosa* infections can include bacterial survival inside epithelial cells. Previously, we showed this involves multiple roles played by the type three-secretion system (T3SS), and specifically the effector ExoS. This includes ExoS-dependent inhibition of a lytic host cell response that subsequently enables intracellular replication. Here, we studied the underlying cell death response to intracellular *P. aeruginosa*, comparing wild-type to T3SS mutants varying in capacity to induce cell death and that localize to different intracellular compartments. Results showed that corneal epithelial cell death induced by intracellular *P. aeruginosa* lacking the T3SS, which remains in vacuoles, correlated with activation of NF-κB as measured by p65 relocalization and TNFα transcription and secretion. Deletion of caspase-4 through CRISPR-Cas9 mutagenesis delayed cell death caused by these intracellular T3SS mutants. Caspase-4 deletion also countered more rapid cell death caused by T3SS effector-null mutants still expressing the TSSS apparatus that traffic to the host cell cytoplasm, and in doing so rescued intracellular replication normally dependent on ExoS. While HeLa cells lacked a lytic death response to T3SS mutants, it was found to be enabled by interferon gamma treatment. Together, these results show that epithelial cells can activate the noncanonical inflammasome pathway to limit proliferation of intracellular *P. aeruginosa*, not fully dependent on bacterially-driven vacuole escape. Since ExoS inhibits the lytic response, the data implicate targeting of caspase-4, an intracellular pattern recognition receptor, as another contributor to the role of ExoS in the intracellular lifestyle of *P. aeruginosa*.

## Author Summary

*Pseudomonas aeruginosa* can exhibit an intracellular lifestyle within epithelial cells in vivo and in vitro. The type three secretion system (T3SS) effector ExoS contributes via multiple mechanisms, including extending the life of invaded host cells. Here, we aimed to understand the underlying cell death inhibited by ExoS when *P. aeruginosa* is intracellular. Results showed that intracellular *P. aeruginosa* lacking T3SS effectors could elicit rapid cell lysis via the non-canonical inflammasome pathway. Caspase-4 contributed to cell lysis even when the intracellular bacteria lacked the entire T33S and were consequently unable to escape vacuoles, representing a naturally occurring subpopulation during wildtype infection. Together, the data show the caspase-4 inflammasome as an epithelial cell defense against intracellular *P. aeruginosa*, and implicate its targeting as another mechanism by which ExoS preserves the host cell replicative niche.

## Introduction

*Pseudomonas aeruginosa* is an important human opportunistic pathogen, able to cause both life- and sight-threatening infections in multiple body sites. While often referred to as an extracellular pathogen, *P. aeruginosa* can adopt an intracellular lifestyle in various epithelial cell types, including corneal [1], bronchial [2], HeLa cells [3] and in vivo [4]. Research published by us and others has shown that the ability of *P. aeruginosa* to persist/replicate inside a host cell depends on the type three secretion system (T3SS), with multiple roles played by the T3SS effector ExoS [5–7] encoded by only invasive strains [8].

ExoS is a bifunctional protein with both RhoGAP and ADP ribosyltransferase (ADPr) activity, and the broad substrate specificity of its ADPr domain [9, 10] enables ExoS to impact multiple host processes [11, 12]. Demonstrated effects of ExoS include inactivation of host cell proliferative/survival signaling, disassembling the host cell cytoskeleton, freezing of intracellular membrane trafficking, and disruption of ROS generation [9, 13–16]. While the molecular components of host cells targeted by ExoS to enable *P. aeruginosa* to persist intracellularly have not yet been elucidated, its ADPr activity is required, and the mechanisms shown include both inhibition of autophagy [17] and evasion of lysosomes [18]. Recently, we reported another role for the ADPr activity of ExoS in intracellular persistence by *P. aeruginosa*; the preservation of host cell viability [19], which was further explored here.

Eukaryotic host cells can generally sense and respond to invasion by intracellular pathogens [20, 21]. Responses can include initiation of regulated cell death pathways [22], which may subsequently be suppressed by the bacteria to preserve their intracellular niche [23]. An example of this death response is pyroptosis, a form of lytic cell death that tends to be inflammatory [20]. Multiple pyroptotic pathways have been identified, each sensing different pathogen-associated molecular patterns (PAMPs) [24–27] or pathogen-associated aberrant activities [28, 29]. The sensor then converges on the activation of a cysteine-aspartic protease: caspase-1 for canonical inflammasome pathways [30, 31], and caspase-4/5 for later-discovered pathways designated noncanonical [32–34]. Inflammatory caspases cleave and activate gasdermin D [35, 36], which assembles into pores in host plasma membranes [37]. This leads to release of cytokines [38], and total lysis for many cell types [39]. Caspase-1 can also process cytokines IL-18 and IL-1β into mature forms [40], which are secreted through gasdermin D pores or released after cell lysis [35].

*P. aeruginosa* effectors ExoS and ExoT can reduce IL-1β secretion in macrophages [41–43], suggesting inflammasome interference might occur. However, responses of macrophages and epithelial cells can differ, and while inflammasome function has been well-studied in macrophages and other myeloid cells, the repertoire of inflammasomes shown in epithelial cells (e.g. corneal epithelial cells) is more limited, and functions less-well understood [44]. One inflammasome pathway studied in some corneal diseases and cultured cells is NLRP3 [45, 46], which can detect numerous stimuli [47] including ionic flux from pore formation [48], lysosomal damaging agents [49], and bacterial RNA [50]. However, not all relevant studies verified which corneal cell type expressed NLRP3, or explicitly determined if NLRP3 was activated, versus other inflammasome pathways that could also yield mature IL-1β secretion. Recently, both caspase-4 (detects cytoplasmic LPS [51]) and NLRP1 (detects pathogenic enzymatic activities and dsRNA [28, 52]) were shown to be expressed and functional in the human corneal epithelium [53, 54]. The Protein Atlas RNAseq data set for the corneal epithelial limbal stem cell line hTCEpi [55] provides evidence for expression of caspase-4, caspase-5 (an additional LPS sensor [33, 56]), NLRP1, and NLRC4 (although its required sensor protein NAIP [57] was not detected) [58]. While NLRP3, Pyrin, and AIM2 inflammasomes were not detected in the hTCEpi cell line, this does not preclude upregulation upon specific stimuli, or in the context of corneal tissue in vivo [58].

Having shown that the T3SS effector ExoS inhibits rapid cell lysis induced by intracellular *P. aeruginosa* [19], in this study we investigated the mechanisms underlying host cell death elicited and modulated by intracellular *P. aeruginosa*. In doing so, we leveraged the finding that *P. aeruginosa* mutants lacking the entire T3SS (Δ*exsA* mutants) remain toxic to corneal epithelial cells [59], contrasting with HeLa and CHO cells [60, 61]. Results showed that caspase-4 is required for corneal epithelial cell death in response to *P. aeruginosa* invasion, and in this way limits accumulation of an intracellular population of bacteria. While caspase-4-dependent death occurred even if bacteria lacked the ability to leave vacuoles/enter the cytoplasm, the response was faster when they could. Moreover, the death response was enabled in otherwise unresponsive (HeLa) cells after stimulation with interferon gamma, which has been shown to limit cytoplasmic sub-populations of *Salmonella* within HeLa cells in a manner dependent on Caspase-4 and GBP proteins [62]. Since ExoS inhibits the cell death response to intracellular *P. aeruginosa*, these results implicate ExoS targeting of a caspase-4 dependent response as another contributor to its well-established role in intracellular survival by *P. aeruginosa*.

## Results

### *P. aeruginosa* lacking the T3SS kill corneal epithelial cells but not HeLa cells.

**Figure 1.**
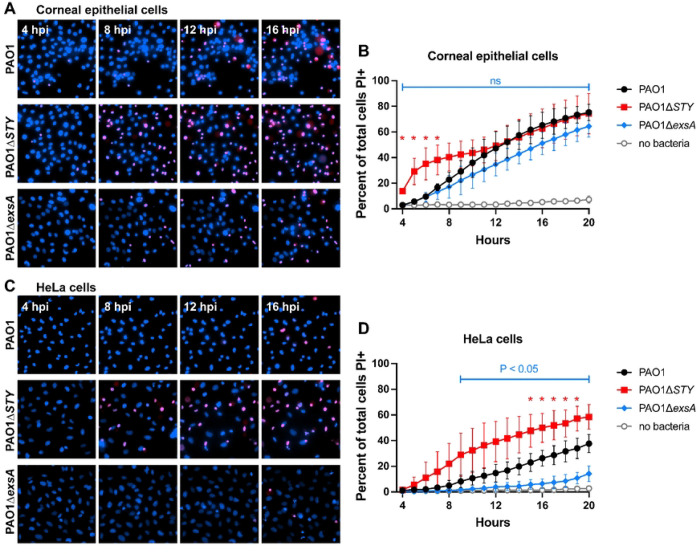
Host cell death rates from *P. aeruginosa* mutant infections. **(A)** Corneal epithelial cells (hTCEpi) were infected with indicated strain: wt PAO1, PAO1Δ*exoSTY*, or PAO1Δ*exsA* for 3 hours at an MOI equal to 10. Hoechst (blue) was added to label nuclei. Non-associated bacteria were removed, and media with amikacin and propidium iodide (red) was added to kill extracellular bacteria. Cells were imaged hourly from 4 to 20 hours post infection. Select fields from indicated times are shown. **(B)** The percent of cells positive for propidium iodide at each timepoint was determined by using a custom FIJI macro to segment all nuclei from the Hoechst channel, and all nuclei of dead cells in the propidium iodide channel. Multiple column t-tests were performed comparing wt PAO1 and PAO1Δ*exsA*, and SD error bars are displayed from three replicates. **(C)** Experiment was performed identically as in panel A, but using HeLa cells. **(D)** Analysis was performed as in panel B, using the data obtained in panel C.

**Figure 2.**
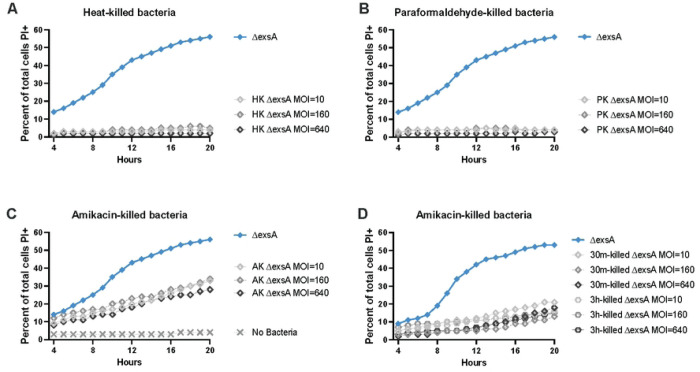
Live bacteria kill corneal epithelial cells. Corneal epithelial cells (hTCEpi) were infected with PAO1Δ*exsA* for 3 hours at an MOI of 10. An identical quantity of bacteria was boiled for 10 minutes prior to infecting cells (**A**), fixed with 2% paraformaldehyde for 10 minutes before infecting cells (**B**), or treated with 1000 μg/ml amikacin for 30 minutes before infecting cells (**C**). MOIs of 160 and 640 were accomplished by adding a greater volume of the same inoculum, which accounts for live bacterial replication from the initial MOI of 10 during the 3-hour infection period. Hoechst (blue) was added to label nuclei. Non-associated bacteria were removed, and media with amikacin and propidium iodide (red) was added. Cells were imaged hourly from 4 to 20 hours post infection. Panels A-C were conducted simultaneously with different bacterial killing methods displayed on separate graphs. (**D**) Experiment conducted as in panel C, however a subset of PAO1Δ*exsA* bacteria were exposed to amikacin for either 30 minutes or 3 hours prior to inoculation of corneal epithelial cells. Cell labeling and analysis was conducted identically to prior panels. Each experiment was conducted twice, and a represented replicate is shown.

Generally, it is thought that *P. aeruginosa* mutants missing the T3SS (i.e., Δ*exsA* mutants) do not cause lytic death of cultured epithelial cells, as shown for HeLa and CHO cells [60, 61]. However, our published work has shown that Δ*exsA* mutants are able to kill corneal epithelial cells, with contributions made by the intracellular population [59]. Recognizing that differences between cell types could potentially assist in deciphering mechanisms, we directly compared corneal epithelial cells (hTCEpi) [55] to HeLa cells using the same methods. Impact of wild type PAO1, Δ*exsA* mutants (lacking the entire T3SS), and Δ*exoSTY* mutants (lacking all known T3SS effectors) were studied. After a 3-hour invasion period, extracellular bacteria were eliminated using the non-cell permeable antibiotic amikacin [3]. Cell death rates were measured with a FIJI macro that counts propidium iodide-positive nuclei (permeabilized cells) and Hoechst-labeled nuclei (total cells) and reports a ratio over a 20-hour post-infection period [19]. The results confirmed that the two cell types were differentially susceptible. Death rates for corneal epithelial cells were statistically similar for wild type and Δ*exsA* mutants (Figure 1A–B), whereas HeLa cells infected with Δ*exsA* mutants showed very little cell death even at the 20-h post infection time point when most corneal epithelial cells had died (Figure 2C–D). In both cell types, the Δ*exoSTY* mutant yielded the most rapid cell death as noted previously [19]. Thus, corneal epithelial cells have an intrinsic response to T3SS-null bacteria that is absent in HeLa cells, which may underly cell death occurring in response to T3SS-positive bacteria.

### Cell death from T3SS-null mutants is driven by live, culturable intracellular bacteria.

In a previous study we demonstrated that the corneal epithelial cell death response to Δ*exsA* mutants depends on intracellular bacteria, using the cell-permeable antibiotic ofloxacin to suppress them [59]. Results showed that host cell death was reduced in a dose dependent fashion by ofloxacin compared to untreated controls. Importantly, in both test and control samples, the extracellular bacteria had already been killed using a non-cell permeable antibiotic before studying the impact of intracellular populations. Still unknown was whether the killed extracellular bacteria contributed to triggering host cell death measured at a later time point. To address this, Δ*exsA* mutants were killed using multiple strategies before challenging cells. Because live bacteria replicate in tissue culture media during the 3-hour invasion period, MOIs that approximated bacterial numbers two or three hours post infection (i.e., 160 or 640) were also tested. Neither heat-killed nor paraformaldehyde-fixed bacteria induced corneal epithelial cell cytotoxicity (Figure 2A, B). However, bacteria killed using antibiotic (amikacin), caused a small but still detectable cell death response that was independent of MOI. (Figure 2C). Potentially explaining that result, visual observation during time lapse configuration revealed a small fraction of amikacin-treated bacteria that continued to display swimming motility (**Supplemental Movie 1**) despite not being recoverable by culturing on TSA agar, suggesting transient persisters retaining some functional capacities remained present in the assay. Bacteria were exposed to amikacin for a longer time period (3 hours), prior to inoculating corneal epithelial cells, although this did not change the experimental outcome (Figure 2D). Thus, extracellular amikacin-killed bacteria may contribute to the death of a sub population of host cells in our experimental system, however live bacteria lead to greater host cell death rates, much of which is attributed to intracellular bacteria [59].

### Dynamics of cell death in only bacterially-occupied cells confirms roles for both T3SS machinery and T3SS effectors.

Visual inspection of infected cells suggested that not all cells were occupied by bacteria, and that some apparently non-occupied cells also died. Recognizing that this could skew results in bulk analyses of cell death rates, we performed additional experiments to consider only bacterially-occupied cells in our analyses. To enable this, we employed methods that allowed simultaneous detection of viable intracellular bacteria and host cell [19]. To monitor timing of lytic cell death we used propidium iodide, which labels the nucleus only if the host cell membrane becomes permeabilized. Wild type (PAO1) was compared to mutants lacking the entire T3SS (Δ*exsA* mutants), and Δ*exoSTY* mutants lacking only T3SS effectors, using quantitative time lapse imaging over 20 h. Since Δ*exsA* mutants do not kill HeLa cells, this analysis was only done using corneal epithelial cells.

To detect Δ*exsA* mutants inside cells, we previously used an arabinose-inducible green fluorescent protein (GFP) expression vector pBAD-GFP [59]. This adaptation of a method originally described for monitoring viability of intracellular *Salmonella* (29) involves induction of GFP expression using arabinose induction only after bacteria have invaded cells and extracellular populations are killed (using amikacin). In an attempt to keep experimental methods consistent across samples, we explored the feasibility of using the induction method to also study wild type and the Δ*exoSTY* mutants. Unfortunately, fewer bacterial cells exhibited cytoplasmic spread using the arabinose induction method compared to our previously used T3SS reporter method (**Supplemental Figure 1A**). Moreover, while the host cell death rate was similar for wild type PAO1 using both methods, it trended lower for Δ*exoSTY* mutants expressing arabinose-induced GFP (**Supplemental Figure 1B)**. This suggested GFP induction in this context impacts the T3SS or some other components of the intracellular pathway relevant to cytoplasmic entry or spread, a step important for intracellular infection by wild type and Δ*exoSTY* mutants, but not for Δ*exsA* mutants (which cannot escape vacuoles due to lack of the T3SS). Indeed, T3SS effector secretion was found reduced *in vitro* using EGTA stimulation in the presence of arabinose only when bacteria were transformed with pBAD-GFP (**Supplemental Figure 1C**). Thus, the T3SS expression plasmid pJNE05 was used to study wild type and Δ*exoSTY* mutants, reserving the arabinose induction pBAD-GFP method for studying the Δ*exsA* mutant which cannot be visualized using the T3SS expression system.

**Figure 3.**
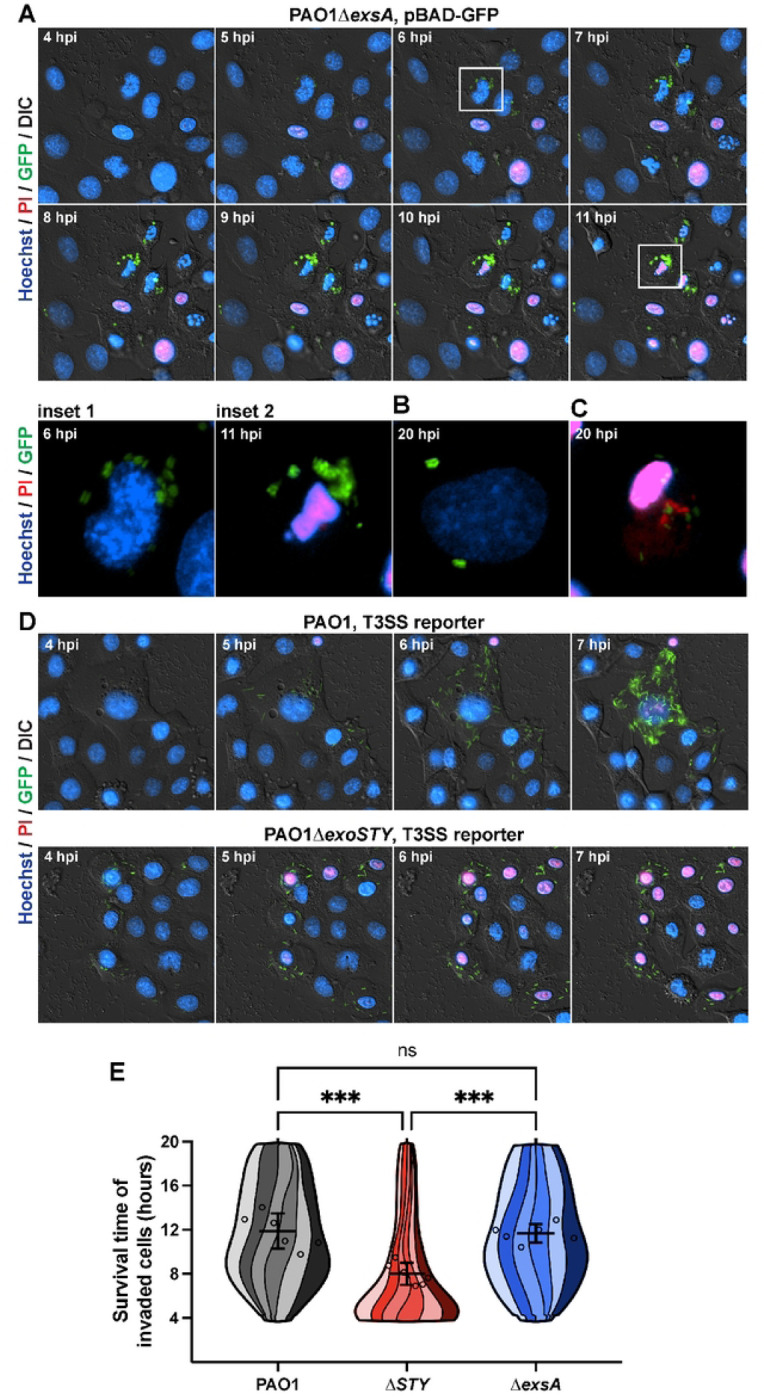
Intracellular Δ*exsA* mutants kill invaded cells at a similar rate to wt PAO1-invaded cells. **(A)** Bacteria were transformed with an arabinose-inducible GFP plasmid. Corneal epithelial cells (hTCEpi) were infected at an MOI of 10 and extracellular bacteria eliminated at 3 hours post infection using amikacin. A final concentration of 1% arabinose was used to induce GFP in surviving intracellular bacteria beginning at 3.5 hours post infection. Time lapse imaging was conducted hourly from 4 to 20 hours, and host cell nuclei detected with Hoechst and propidium iodide to determine time of death. Insets 1–2 show the boxed cell at 6 or 11 hours post infection, respectively. **(B)** An example of a live invaded cell from 20 hours post infection. **(C)** Example of a dead invaded cell from 20 hours post infection, where the PI channel has been saturated such that PI-positive bacteria can be visualized. **(D)** The T3SS-GFP reporter pJNE05 was used to visualize wt PAO1 and PAO1Δ*exoSTY* infections, as described previously [19], to compare to PAO1Δ*exsA* infections. Time lapses from panels A through D are available as **Supplemental Movie 2**. **(E)** A computational analysis approach was used to segregate only invaded cells, and determine their survival time in hours. The survival times of all invaded cells from six replicates were combined into a single super violin plot. Mean survival times with SD error bars are displayed, and significance determined by One-way ANOVA. *** P < 0.005.

Images from a typical 20 h time-lapse experiment using Δ*exsA* mutants are shown in Figure 3A. As expected, Δ*exsA* mutants localized to vacuoles inside cells [59]. Most bacteria-occupied cells died before the end of the 20 h assay, as shown by propidium iodide labeling of the nucleus. On the other hand, Figure 3B shows an invaded cell still alive at 20 hours (no propidium iodide labeling), representing a minority of cells at this time point. Videos of these time lapse experiments are available in **Supplemental Movie 2**, which includes cells visualized in Figures 3B and 3C at 20 hours post infection. As expected, both wild type PAO1 and Δ*exoSTY* mutant infected cells (shown in Figure 3D) replicated in the host cell cytoplasm after bacterial escape from vacuoles [19]. In both cases, most occupied cells died by the end of the assay, the Δ*exoSTY* mutant doing so even more rapidly, expected due to lack of ExoS which normally counters cell death [19]. Importantly, propidium iodide labeling (lytic cell death) preceded the loss of the GFP signal from intracellular bacteria, which was replaced with propidium iodide positive bacterial bodies (Figure 3C and **Supplemental Movie 2)**. Given that lytic cell death allows the non-cell permeable antibiotic used in these assays to enter cells, the bacterial GFP signal loss shortly after cell lysis confirmed that labeled bacteria had been inside a cell with an intact plasma membrane, and were not simply antibiotic resistant extracellular bacteria.

A computational approach was used to quantitatively assess the timing of when populations of invaded cells died [19], and a super violin plot visualization script developed by Kenny and Schoen was used to show biological replicates in single plots [63]. This analysis confirmed quantitatively that intracellular Δ*exsA* mutants killed their occupied cells at a broad range of time points. However, the mean value of invaded cell survival times was unexpectedly similar to wild type PAO1 (Figure 3E). The analysis also confirmed that the intracellular Δ*exoSTY* mutant infections caused cell lysis at a more rapid rate, significantly different from both wild type PAO1 and Δ*exsA* mutants [19]. Thus, while cell death can be driven by vacuolar contained bacteria, it was more rapid if they could access the cytoplasm while lacking effectors/ExoS able to counter cell death.

### T3SS-null mutant bacteria engage NF-κB signaling in corneal epithelial cells.

**Figure 4.**
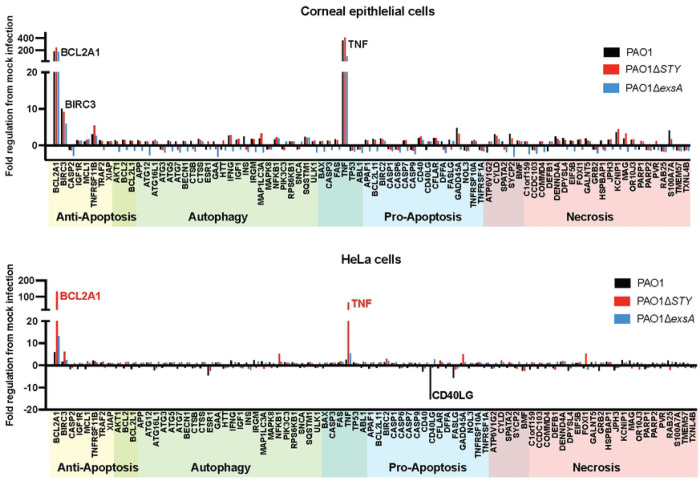
Transcriptional responses of infected host cells. **(A)** Corneal epithelial cells (hTCEpi) were infected with wt PAO1, PAO1Δ*exoSTY*, or PAO1Δ*exsA* for 3 hours at an MOI equal to 10. Non-associated bacteria were removed, and media with amikacin was added for 1 additional hour. At four hours, RNA was purified from infected cells, and RT PCR analysis performed using a commercial array for genes associated with specific cell death pathways. **(B)** Experiment performed identically to panel A, except using HeLa cells.

To explore molecular mechanisms involved in *P. aeruginosa*-driven cell death in these assays, a real time PCR array with selected genes involved in regulated cell death pathways was used to study candidate host responses. Corneal cells and HeLa epithelial cells were infected with wild type PAO1, Δ*exoSTY* mutants or Δ*exsA* mutants. Results showed that TNFα and BCL2A1 were upregulated in excess of fifty-fold in both cell lines: (Figure 4). In the corneal cells, wild type and both mutants upregulated these genes, while in HeLa cells, only the PAO1Δ*exoSTY* mutant had that impact.

**Figure 5.**
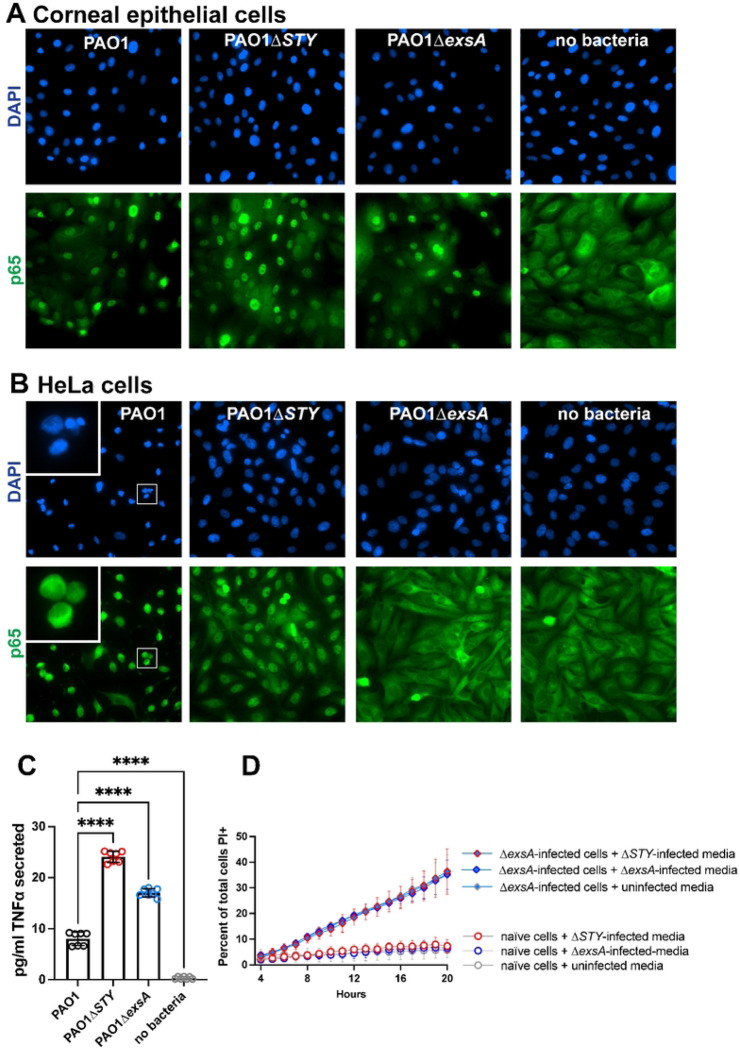
NF-κB signaling and the role of secreted factors in cell death. **(A)** Corneal epithelial cells (hTCEpi) were infected with wt PAO1, PAO1Δ*exoSTY*, or PAO1Δ*exsA* for 3 hours at an MOI equal to 10. Then media was replaced with amikacin-containing media. Cells were fixed at 4 hours post infection and p65 localization was determined using immunofluorescent staining. Nuclei were labeled with DAPI. **(B)** Experiment performed identically to panel A, using HeLa cells. **(C)** Corneal epithelial cells were infected as described in panel A. Supernatant of infected cells was collected at 4 hours post infection and TNFα measured by ELISA. **(D)** Supernatants of corneal epithelial cells infected with PAO1Δ*exoSTY*, PAO1Δ*exsA*, or uninfected were collected at 4 hours post infection and sterilized with 0.22 μm filter, and used as the replacement media on uninfected cells or PAO1Δ*exsA* infected cells at 3 hours post infection, combined with amikacin and propidium iodide. Hoechst was used to label all nuclei. The cell death rates were measured by time lapse imaging, error bars display SD from three replicates.

Since TNFα and BCL2A1 can be regulated by NF-κB signaling, activation of NF-κB was measured at the single-cell level by observing translocation of p65 to the nucleus (Figure 5A). In corneal epithelial cells, infection with each tested strain caused p65 translocation, correlating with positive target gene transcription. In HeLa cells, only infection with Δ*exoSTY* mutants led to p65 translocation (Figure 5B). The result with wild type PAO1 infection was difficult to discern due to cell rounding; however, p65 appeared excluded from the nuclear portion of HeLa cells (Figure 5B, inset). Overall, nearly every cell in the population showed similar p65 localization, despite fewer than half of cells being invaded in this experimental system [3]. This finding correlated with the transcriptional data, and is suggestive of a mechanism in which the T3SS effectors limit p65 translocation in HeLa cells, but not corneal epithelial cells.

Since TNFα is associated with both apoptotic and necrotic cell death, TNFα secretion from infected cells was measured by ELISA. The results showed that while wild type PAO1 stimulated TNFα secretion from corneal epithelial cells, higher levels were detected after infection with the Δ*exoSTY* mutant, and the Δ*exsA* mutant causing an intermediate response (Figure 4B).

Having found TNFα secretion levels correlated with cell death rates, we next asked if there was a direct causative relationship that might also explain why some cells without visible intracellular bacteria could also be killed. Thus, the media from naïve cells or Δ*exsA* mutant-infected cells was replaced with filter-sterilized media from Δ*exoSTY* mutant-infected cells (high TNFα). The outcome showed that this did not enhance host cell death rates (Figure 4C), suggesting cell death is not driven by a secreted factor such as TNFα, and requires extracellular bacteria, at a minimum (Figure 2).

### Caspase-4 is involved in cell death response to intracellular *P. aeruginosa*.

The morphology of dying cells included membrane integrity loss, intact nucleus, and transient blebbing, all suggestive of pyroptosis (Figure 5) [22]. Since caspase-4 can restrict numerous other Gram-negative bacteria [62, 64], and the pathogen *Shigella* inhibits the caspase-4 inflammasome by multiple mechanisms to survive [65–67], and corneal epithelial cells were shown to express caspase-4 [54], we tested the hypothesis that caspase-4 was involved in driving cell death in response to intracellular *P. aeruginosa*. We generated *CASP4* knockout corneal epithelial cells using CRISPR-Cas9, and loss of caspase-4 protein was confirmed by Western blot (**Supplemental Figure 2**). Control cell lines with non-targeting guide RNA were also generated using an identical selection strategy and grown up as monoclonal lines; each exhibited death rates consistent with wild type, non-transduced hTCEpi cells (**Supplemental Figure 3**).

**Figure 6.**
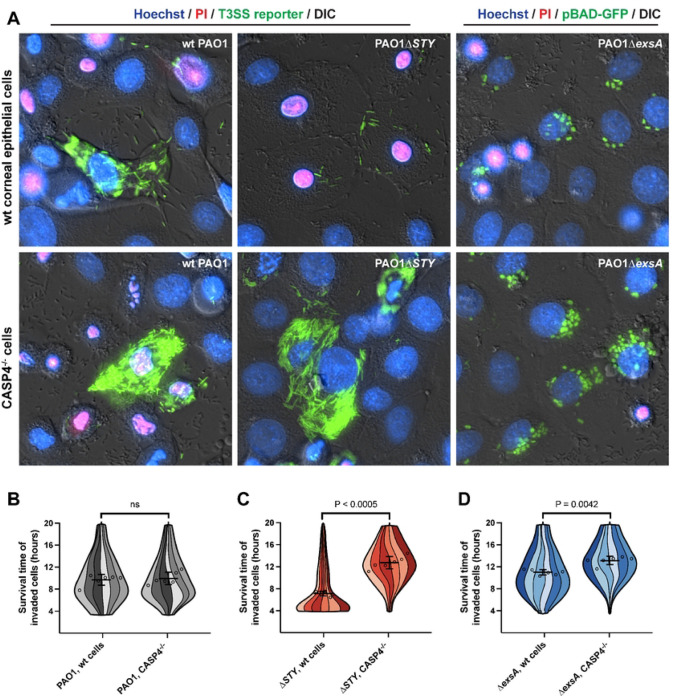
Caspase-4 limits intracellular replication and persistence of T3SS mutant bacteria. (A) Corneal epithelial cells (hTCEpi) or cells knocked out for caspase-4 were infected with wt PAO1, PAO1Δ*exoSTY* (each visualized by T3SS-GFP reporter) or PAO1Δ*exsA* (pBAD-GFP induced) at an MOI of 10. Nuclei were labeled with Hoechst and Propidium iodide. Extracellular bacteria were eliminated with amikacin at 3 hours post infection, and imaged hourly from 4 to 20 hours post infection. Images from 8 hours post infection are shown. Full-field time lapses are available as **Supplemental Movie 3**. (**B-D**) Cells from wt PAO1 (B), PAO1Δ*exoSTY* (C), or PAO1Δ*exsA* (D) infections were analyzed with a computational approach to segregate only invaded cells and measure survival times in hours. Six replicates were combined into a single super violin plot. Mean survival times with SD error bars are displayed, and significance determined by Student’s t-test.

Invasion by wild type PAO1 yielded similar cell death timing in both *CASP4* knockout cells as wild type corneal epithelial cells (Figure 6A, B). In contrast, Δ*exoSTY* mutant-invaded cells experienced significantly increased cell survival times, which subsequently allowed substantial further accumulation of intracellular bacteria (Figure 6A, C). A smaller but significant increase of invaded cell survival times were also observed with PAO1Δ*exsA* mutant-occupied *CASP4* knockout cells (Figure 6D). These data implicated the non-canonical inflammasome as a dominant response to cytoplasmic invasion of T3SS-positive *P. aeruginosa* independently of the T3SS effectors, and also show involvement in responding to vacuole-occupying *P. aeruginosa* that do not express the T3SS. This finding that cell death rates for wild-type PAO1 remain unchanged in *CASP4* knockout cells aligns with our previously published data showing that the T3SS effector exotoxins, specifically the ADP ribosyltransferase activity of ExoS [19], counters cell death. Of note, while *CASP4* knockout reduced corneal epithelial cell death associated with intracellular Δ*exsA* mutants, many of the cells still died by the end of the 20-hour imaging time frame (Figures 1 and 6D). This suggests additional mechanisms for recognizing vacuolar/T3SS-negative *P. aeruginosa* in addition to caspase-4, and that they are also absent from HeLa cells.

### IFN-γ-stimulation enables HeLa cells to respond to intracellular *P. aeruginosa*.

**Figure 7.**
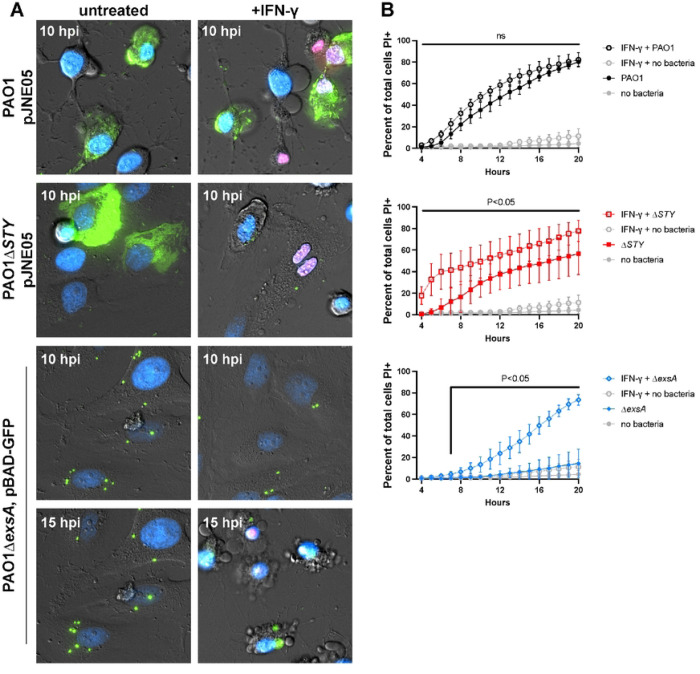
IFN-γ-stimulated HeLa cells are not permissive for PAO1Δ*exoSTY* intracellular replication nor PAO1Δ*exsA* intracellular persistence. **(A)** HeLa cells were treated with 50 ng/ml IFN-γ for 16 hours prior to infection with wt PAO1, PAO1Δ*exoSTY* (each using the T3SS-GFP reporter) or PAO1Δ*exsA* (pBAD-GFP induced) at an MOI of 10. Nuclei were labeled with Hoechst and Propidium iodide. Extracellular bacteria were eliminated with amikacin at 3 hours post infection, and imaged hourly from 4 to 20 hours post infection. Select time frames are shown at either 10 or 15 hours post infection. Full-field time lapses are available as **Supplemental Movie 4**. **(B)** The rate of cell death was determined using host cell nuclear stains. All panels are from the same experiment with only indicated bacterial strain shown. Error bars show SD from three replicates. Multiple column t-tests were performed comparing IFN-γ-treated cells to untreated.

HeLa cells can support cytoplasmic hyper-replication of *P. aeruginosa* before significant impact on host cell viability occurs [3, 19]. The bacterium *Salmonella* accomplishes a similar feat [68]. However, Santos and colleagues demonstrated that IFN-γ stimulation causes HeLa cells to lyse in response to *Salmonella* entry into the cytosol, which was associated with upregulation of GBP proteins and stimulation of the caspase-4 inflammasome. [62]. Here, we examined the impact of IFN-γ stimulation on the HeLa cell response to intracellular *P. aeruginosa*. HeLa cells were treated with 50 ng/ml IFN-γ for 16 hours prior to infection and compared to untreated cells (Figure 7). The kinetics of wild type PAO1-induced host cell death remained unchanged. However, the rate of cell death increased for both PAO1Δ*exoSTY* and for PAO1Δ*exsA* infections (Figure 1 and 7). [60]; IFN-γ-treated HeLa cells occupied by Δ*exsA* mutants died over varied timepoints during the 20 hours of infection similar to results for corneal epithelial cells. Aligning with the quick timing of cell lysis, PAO1Δ*exoSTY* mutants were no longer able to replicate effectively in the cytoplasm of IFN-γ-treated HeLa cells. Thus, IFN-γ-treated HeLa cells resembled corneal epithelial cells in their response to intracellular *P. aeruginosa*. This suggests that the difference between corneal epithelial cells and HeLa cells observed here may be among interferon stimulated genes (such as GBP1-4 in unstimulated cells), or the ability of corneal epithelial cells to promptly upregulate components of the caspase-4 pathway in response to detection of extracellular *P. aeruginosa*, prior to invasion (Figure 4).

## Discussion

Invasive (ExoS-expressing) strains of *P. aeruginosa* can thrive inside a wide range of host cells [2, 3, 5]. After invasion of an epithelial cell, ExoS plays multiple roles in supporting intracellular survival and replication, including inhibition of the host cell death response [19]. Here, we aimed to understand the underlying cell death response inhibited by ExoS.

*P. aeruginosa* is highly adaptable and can adopt diverse phenotypes even in the same environment. Previously, we used corneal and bronchial epithelial cells to show that after invasion, members of the T3SS-positive population escape vacuoles to colonize the cytoplasm while T3SS-off subpopulation members remain in vacuoles [59]. Because vacuoles containing T3SS-off *P. aeruginosa* can become acidified, we originally assumed they were destined for degradation in lysosomes [18]. However, we recently reported that vacuole-bound/T3SS-off populations can report gene expression, including biofilm-associated genes, display enhanced antibiotic resistance, and contribute to triggering host cell death [59]. These characteristics were attributable to absence of the T3SS, with mutants lacking the T3SS (Δ*exsA* mutants) faithfully replicating the phenotype.

Knowing *P. aeruginosa* diversifies intracellularly into vacuole- and cytoplasm- occupying populations differing transcriptionally, that bacteria in both locations trigger cell death, that their locations are determined by T3SS expression state, and that triggered host cell death can be suppressed by the T3SS effector (ExoS), we used T3SS mutants trafficking to different locations to interrogate mechanisms. Results using corneal epithelial cells showed that the death response to intracellular *P. aeruginosa* lacking T3SS effectors (but expressing the T3SS apparatus) involved caspase-4, its deletion inhibiting the rapid pyroptotic lysis. It also partially reduced slower cell death caused by bacteria lacking the entire T3SS that were restricted to vacuoles (Δ*exsA* mutants). We further found that while HeLa cells naturally lack this host cell death response to intracellular *P. aeruginosa*, it could be induced by IFN-γ, a stimulator of numerous genes including factors to assist in activation of the caspase-4 inflammasome [62]. Given that ExoS expressed by wild-type *P. aeruginosa* inhibits the cell death response to intracellular bacteria, these findings implicate ExoS-mediated interference with the non-canonical inflammasome pathway/pyroptosis.

Since *P. aeruginosa* is often assumed an extracellular pathogen, previous studies of triggers or manipulation of host epithelial cell death have been from the perspective of extracellular bacteria. In that regard, ExoS has pro-cytotoxic activities. HeLa cells can undergo apoptotic cell death based on both caspase-3 [60] and caspase-8 activity [69], and activation of apoptosis through JNK1 and cytochrome c release [70]. This was also shown using a mammalian expression system for introducing ExoS in the absence of bacteria, which showed it was sufficient to induce HeLa cell apoptosis even in the absence of bacterial interaction [71]. Placing our findings into the context of these earlier HeLa cell studies, we note differences in our experimental systems: while they explored mechanisms for cell death caused by ExoS, we studied cell death triggered by live bacteria inside cells when they lack ExoS. Were intracellular bacteria present in these assays, the HeLa cells used would not have responded to them without stimulation. Our experiments also provide an analysis of only invaded cells excluding extracellular bacteria to focus on impact of intracellular bacteria, and used corneal cells which naturally respond to them. We then showed that IFN-γ-stimulated HeLa cells respond in the same manner: ExoS inhibits rather than drives a host cell death response. In sum, while introducing ExoS in isolation is a useful surrogate for *in vitro* experimentation to address specific questions, other bacterial factors/live bacteria can modify impact in other situations. Our data also highlight the value of using cell lines that have not been substantially transformed, and imaging studies to put into perspective important information resulting from biochemical analyses. Despite differences in experimental models, it remains important to reconcile outcomes, and the innate ability of ExoS to both drive and inhibit cell death, involving different types of regulated cell death, and correlating with presence or absence of internalized *P. aeruginosa,* is interesting and worth exploring. Indeed, it could aid in determining mechanisms by which ExoS blocks pyroptosis, given crosstalk between pyroptosis and apoptosis noted in other cell types [72].

While HeLa cells are reported to express caspase-4, results presented in this report showed that activation in this context requires upregulation of IFN-γ stimulated genes, which include GBP proteins that can bind and assist in presenting LPS [62]. It remains unknown if GBP proteins are present or efficiently upregulated in corneal epithelial cells upon exposure to *P. aeruginosa*, however detection of LPS is how other Gram-negative intracellular bacteria such as *Shigella* trigger caspase-4. While our results with Δ*exsA* mutants raises questions about how LPS is detected by factors in the cytoplasm when bacteria remain in vacuoles, proposed mechanisms in other studies include outer membrane vesicle delivery [73] or by HMGB1 [74]. Our findings in this regard are consistent with the observations of others who showed caspase-11 or caspase-5 activation in response to *P. aeruginosa*. [56, 75].

Interaction of GBP proteins (specifically, GBP2) with *P. aeruginosa* for efficient caspase-11 activation has been shown by Balakrishnan et. al*,* interestingly, in the context of T3SS-negative cytotoxic bacteria (ExoU-producing) that are internalized by macrophages [75]. Usually, ExoU encoding strains do not invade cells because T3SS injection from outside the cell causes rapid cell death [76]. Although wild type *P. aeruginosa* is not known to replicate inside macrophages in the same manner as in epithelial cells [7], the authors found that elimination of caspase-11 in mouse BMDMs permitted intracellular proliferation of PopB mutants [75]. Despite differences between the study by Balakrishnan et. al. and our experimental system, the importance of caspase-4/11 in restricting intracellular *P. aeruginosa* niches is consistent. This supports the possibility that ExoS could sustain intracellular niches by targeting these pathways in a broad range of cell types and infection sites.

The specific mechanism by which the ADP-r activity of ExoS interferes with caspase-4-mediated host cell lysis remains to be determined. Recently, *Shigella flexneri* was shown to block the non-canonical inflammasome by two separate mechanisms: caspase-4 inactivation by a newly-identified catalytic activity termed ADP-riboxanation by the effector OspC3 [65, 66], and ubiquitylating gasdermin D with IpaH7.8 to target it for degradation [77]. ExoS is unique among bacterial ADP ribosyltransferases for having numerous host substrates, and currently none have been shown to interact specifically with the caspase-4/11 pathway [78]. Since there are many possibilities to investigate, comprehensive identification of uncharacterized ExoS substrates and their potential involvement will require a separate study. Considering the T3SS apparatus of extracellular *P. aeruginosa* can trigger cell death via NLRC4 [79], and that *CASP4* knockout does not fully prevent the cell death triggered by vacuole-bound T3SS mutants (Figure 6), it would also be worth exploring if additional cell death pathways are impacted by ExoS.

In summary, the results of this study demonstrate a role for caspase-4 in limiting intracellular colonization by *P. aeruginosa*, a phenomenon triggered by the presence of intracellular bacteria but inhibited by the T3SS effector ExoS. Together with research previously published by us and others using corneal, bronchial, and stimulated HeLa cells, this suggests the following sequence of events for the intracellular lifestyle of invasive *P. aeruginosa:* bistable T3SS expression upon host cell contact, invasion of T3SS-negative bacteria, cytoplasmic access associated with intracellular T3SS activation, triggering of caspase-4 mediated pyroptosis, and inhibition of both pyroptosis and autophagy by ExoS.

While providing more information about *P. aeruginosa*-host epithelial cell interactions, the data presented in this report illustrate the value of quantitative imaging in teasing apart complexities for a pathogen as versatile as *P. aeruginosa*. They also highlight the importance of appreciating differences between cell types, including results showing a relevant mechanism lacking in a cell line can remain a useful experimental system with host response factors that can be toggled on and off using alternate means of stimulation (IFN-γ, in this instance). Importantly, the data continue to support the notion that *P. aeruginosa* can function as both an intracellular and extracellular pathogen, suggestive of a T3SS effector function devoted to inhibition of an *intracellular* pattern recognition receptor, consistent with its unusual capacity to adapt to its environment, survive hardship, and exist ubiquitously. Ultimately, development of effective strategies to prevent or treat the devastating infections caused by *P. aeruginosa* will require a thorough appreciation of these complexities.

## Methods

### Bacterial strains, cell lines, and reagents.

*P. aeruginosa* strain PAO1 (a strain with positive T3SS expression) and isogenic deletions of *exsA*, or ExoS/ExoT/ExoY were used for all infection experiments [80]. Bacteria were grown at 37°C on tryptic soy agar. Plasmids used for visualization were pJNE05 [81] and pBAD-GFP [59]. Plasmid selection was achieved with 100 μg/ml gentamicin. Corneal epithelial cells (hTCEpi) [55] were maintained in complete KGM-2 media (Lonza), lacking gentamicin. All experiments were conducted on cells between passage 49 and 70. HeLa cells were maintained in phenol red-free DMEM (Gibco) with 10% FBS.

### CRISPR-Cas9 knockout cell lines.

Lentiviral particles for transduction were generated from 293T cells transfected with psPAX2 (Didier Trono, Addgene plasmid # 12260 ; http://n2t.net/addgene:12260 ; RRID:Addgene_12260) and pMD.2 (Didier Trono, Addgene plasmid # 12259 ; http://n2t.net/addgene:12259 ; RRID:Addgene_12259), combined with either Cas9 expression vector plenti-Cas9-blast (Feng Zhang, Addgene plasmid # 52962 ; http://n2t.net/addgene:52962 ; RRID:Addgene_52962) [82] or a guide RNA vector plenti-LIC-Puro which was adapted for ligation-independent cloning (kindly gifted by Moritz Gaidt) [83]. Guide RNA sequences targeting caspase-4 (5’- CCACAGAAAAAAGCCACTTA - 3’) or a non-targeting control sequence (5’- GACGGAGGCTAAGCGTCGCA - 3’) were cloned into plenty-LIC-Puro using ligation independent cloning. Six hours after transfection, media on 293T cells was replaced with KGM-2. Media was collected at 48 hours and passed through a 0.45 μm filter and added directly to hTCEpi cells, which were then centrifuged at 1200g for 90 minutes at 37°C. Antibiotic selection with blasticidin (50 μg/ml) or puromycin (10 μg/ml) was performed after two days. For generation of monoclonal lines, cells were lifted by trypsin and seeded as single cells in 96-well plates. Isolated colonies were identified and grown up over approximately 12 days before transfer into 6-well plates to avoid differentiation induced by crowding or confluence. *CASP4* knockout candidates were screened by Western blot: lysates from a 6-well plate were collected in 150 μl RIPA buffer, frozen and thawed, and then clarified by centrifugation for 20 minutes at 4°C. Samples were run on 10% stain free gels (Bio-Rad) and total protein visualized as a loading control prior to transfer to 0.2 μm PVDF membrane. Membrane was blocked with Bio-Rad commercial block for 10 minutes, and probed with anti-caspase-4 antibody (Santa Cruz, sc-56056, 1:1000 or 100 ng/ml) overnight at 4°C with rocking. Blot was washed using TBST, and antibody detected with goat-anti-rabbit HRP (Bio-Rad, 1706515, 1:5000).

### Infection experiments.

Corneal epithelial cells were plated on No. 1.5 glass bottom 24 well plates (MatTek) in KGM-2 with 1.15mM total calcium induce differentiation [55]. Corneal cells were plated at 75% confluence the day prior to experiments. HeLa cells were maintained in DMEM with 10% FBS and plated at 50% confluence the day prior to experiments. For imaging, HeLa cells were plated on optical plastic 8-well chambered coverslips (ibidi). Where indicated, 50 ng/ml IFN-γ (Peprotech) was added at 16 hours prior to infections of HeLa cells and maintained throughout the infection and imaging.

Bacterial suspensions were made in PBS from 16-hour lawns grown on TSA media at 37°C. The optical density at 540 nm was measured, and generally ranged from 1–4. An MOI of 10 was calculated using OD540 of 1 equal to 4 × 10^8^ CFU per mL. Bacteria (volumes typically 0.5–4 μl) were directly added to cell culture media and allowed to infect for 3 hours. For live imaging experiments, Hoechst (0.8 μg/ml) was added to the cells prior to infections and allowed to label the cells during the 3-hour infection period. Then media was removed, and replaced with media containing the antibiotic amikacin (200 μg/ml) and propidium iodide (0.75 μg/ml). Where needed, a 1:10 dilution of 10% arabinose in either KGM-2 or DMEM was added at 3.75 hours post infection, which induced GFP expression in the protected population of intracellular bacteria carrying the pBAD-GFP plasmid.

### Bacterial killing.

Aliquots of bacterial inocula were measured by spectrophotometry and volumes to be added to achieve effective MOIs of 10, 160, or 640 were determined as above. Heat-killed bacteria were boiled for 10 minutes. Paraformaldehyde-killed bacteria were fixed in a 2% solution of paraformaldehyde in PBS for 10 minutes, quenched with 150mM glycine for 10 minutes, and washed in PBS using centrifugation. Amikacin-killed bacteria were exposed to 1000 μg/ml amikacin for 30 minutes or 3 hours prior to adding to cells. All bacteria were plated on TSA agar in 5 μl volumes to ensure complete killing.

### RT-PCR arrays.

Cells in 10cm dishes were infected with PAO1, PAO1Δ*exoSTY*, or PAO1Δ*exsA* for 3 hours at an MOI of 10, and media was replaced for 1 hour with 200 μg/ml amikacin. At 4 hours, cells were washed twice in PBS and collected in 1ml TriReagent (Sigma). RNA was collected using Zymo Direct-zol RNA MiniPrep with in-column DNAse digestion for an extended time of 40 minutes. RNA quality was assessed by BioAnalyzer at QB3 Genomics, UC Berkeley, Berkeley, CA, RRID:SCR_022170. cDNA synthesis using 0.5μg of RNA was performed with the RT2 first strand kit (Qiagen) according to manufacturer’s instructions. The RT^²^ Profiler^™^ PCR Array Human Cell Death PathwayFinder (PAHS-212Z, Qiagen) product was selected, and RT PCR performed on a Roche LightCycler 96 instrument. Analysis was performed using Qiagen’s Data Center Analysis Portal. (https://geneglobe.qiagen.com/us/analyze). Graphs of results were prepared using Graph Pad Prism 9.

### Fixed immunostaining.

Cells were seeded on No. 1.5 glass coverslips and infected as described above. At four hours post infection, cells were washed twice in PBS and fixed in 4% paraformaldehyde in PBS for 10 m. Cells were washed twice in PBS, and free aldehydes neutralized with 150 mM glycine in PBS for 10 minutes. Then cells were washed twice in PBS, and permeabilized and blocked (5% FBS, 2.5% cold fish skin gelatin, 0.1% TritonX-100, 0.05% Tween-20 in PBS) for 1 hour. Then primary antibody was added in antibody solution (2.5% FBS, 1.25% cold fish skin gelatin, 0.1% TritonX-100, 0.05% Tween-20 in PBS) overnight at 4°C. Cells were washed 4 times (5 minutes per wash), and secondary antibodies were added in antibody solution for 1 hour in darkness. Cells were washed once, labeled with DAPI for 5 m, and washed two more times (5 minutes each). Coverslips were mounted on ProLong Diamond and cured overnight prior to imaging.

### Microscopy.

Images for Figs 1, 3, and 5 were captured on a Nikon Ti-E inverted microscope equipped with a Lumencor Spectra X LED Light Engine illumination source. All other images were captured on a Ti2-E inverted microscope base with X-Cite XYLIS XT720S Broad Spectrum LED Illumination System. Both systems used Nikon perfect focus, an Okolab stage-top incubation chamber, a DS-Qi2 CMOS camera, CFI Plan Apochromat Lambda D 40X air NA 0.95 objective for time lapse images, and CFI Plan Apochromat Lambda D 60X Oil NA 1.42 objective for fixed slides. For time lapses, fields were selected between hours 3 and 4 without observation of fluorescence channels to avoid bias. Eight fields per condition were imaged hourly from 4 to 20 hours post infection.

### Image Analysis.

Time-lapse images were computationally analyzed using two custom macros written for the FIJI package of Image J, with final results table encoded as intensity values in a TIF image, which was previously described [19]. The code for measuring rates of cell death for the whole population, and set of macros for tracking the invasion state of cells is available in a GitHub repository: https://github.com/Llamero/Nuclei_analysis-macro. Subsequent Python scripts for exporting the analysis TIF file and processing it are available in the following GitHub repository: https://github.com/abbykroken/cell_survival_with_bacteria.

### In vitro T3SS effector secretion.

Bacteria were grown for 5 hours in 5ml LB media supplemented with 100mM MSG and 1% glycerol at 37°C with shaking at 200 rpm. A concentration of 2mM EGTA was added to induce T3 secretion. Final concentrations of 1% or 0.1% arabinose were added from a 10% stock made in LB media with MSG and glycerol. OD 540 nm readings were taken prior to supernatant protein concentration and used to normalize sample volumes. Bacteria were centrifuged at 12000g to clarify 1ml of supernatant. Proteins were concentrated using TCA precipitation: to 1ml of supernatant, 250 μl of 100% cold TCA was added for 30 minutes, and then centrifuged at 14000g for 5 minutes. The pellet was washed with 1ml of cold acetone twice, briefly dried, and suspended in 4x laemmli buffer based on the starting OD (max volume of 50 μl). Proteins were visualized using 10% stain-free gels (Bio-Rad).

### Statistics.

Statistical analyses were performed and data presented using Graph Pad Prism 9. Super violin plots were prepared using scripts published by Kenny and Schoen [63] and output images aligned on axes generated in Graph Pad Prism 9. Data were shown as a mean ± standard deviation (SD) of 3–6 independent experiments unless otherwise indicated. Comparison of two groups was performed by Student’s t-test, three or more groups by One-way ANOVA with Tukey’s post-hoc analysis. Comparison between two groups for total cell death rates overtime was performed by multiple column t-tests for each timepoint, and the two samples that were compared are specified in the figure legends. In each instance, * P < 0.05, ** P < 0.01, *** P < 0.005 and **** P < 0.001.
